# SARS-CoV-2 samples may escape detection because of a single point mutation in the N gene

**DOI:** 10.2807/1560-7917.ES.2020.25.39.2001650

**Published:** 2020-10-01

**Authors:** Katharina Ziegler, Philipp Steininger, Renate Ziegler, Jörg Steinmann, Klaus Korn, Armin Ensser

**Affiliations:** 1 Institute of Virology, University Hospital Erlangen, Friedrich-Alexander-University Erlangen-Nuremberg (FAU) Erlangen, Germany; 2 Institute of Clinical Hygiene, Medical Microbiology and Infectiology, Paracelsus Medical University, Nuremberg, Germany

**Keywords:** SARS-CoV-2, diagnostic quantitative RT-PCR, single nucleotide polymorphism

## Abstract

We found that a single nucleotide polymorphism (SNP) in the nucleoprotein gene of severe acute respiratory syndrome coronavirus 2 (SARS-CoV-2) from a patient interfered with detection in a widely used commercial assay. Some 0.2% of the isolates in the EpiCoV database contain this SNP. Although SARS-CoV-2 was still detected by the other probe in the assay, this underlines the necessity of targeting two independent essential regions of a pathogen for reliable detection.

The reliable diagnosis of infectious diseases has been greatly facilitated by sensitive and quantitative PCR methods that can detect few and even single genomes of pathogens. However, the high specificity of the assays has the consequence that few or even single nucleic acid changes can substantially compromise sensitivity. This has been demonstrated in several instances, including our own contributions to HIV and influenzavirus assays [1-3]. Here, existing awareness in our diagnostic laboratory prompted a deeper investigation in a particular case of a severe acute respiratory syndrome coronavirus 2 (SARS-CoV-2) infection detected on 21 July that showed a discrepant qRT-PCR test result.

## Case description

A female patient in her 50s presented in mid-July at the city hospital (Day 1) because a close family member had been notified of a positive SARS-CoV-2 test the day before. The close family member had developed cough and fever 6 days earlier, 4 days after both had returned after staying in Romania for several weeks. Our patient had no initial symptoms. 

A nasopharyngeal swab tested with the Xpert Xpress SARS-CoV-2 (GXP) assay (Cepheid Inc., Sunnyvale, United States (US)) was reported as SARS-CoV-2 presumptive-positive with a cycle threshold (Ct) value of 22.7 for the E gene, but a negative result for the N2 gene. For confirmation, the sample was reanalysed with the Allplex SARS-CoV-2 assay (Seegene Inc., Seoul, South Korea) [4,5], which revealed positive results for all three targets (E gene Ct = 23.9, RdRp gene: Ct = 25, N gene: Ct = 27). We performed additional real-time PCR tests according to the Charité protocol [6], which were positive for the E gene (Ct = 25.5) and RdRp (Ct =26.4). 

The patient was released into home isolation. On a follow-up visit 2 weeks later, she reported transient muscular pain but no fever, and her chronic cough from bronchial asthma and smoking had not exacerbated. A nasopharyngeal swab taken at that time also showed a discrepancy between the results of the E gene and N gene in the GXP assay (E gene: Ct = 34.6, N gene: negative). On Day 29, the patient reported olfactory loss and ageusia.

## Ethical statement

Written consent was obtained from the patient for publication in an anonymised manner. Ethical approval was not sought because as the patient was seen in the hospital in the normal course of medical treatment, not within the context of a study.

## Characterisation of mutations in the assays target region

Because of the initial failure of the GXP assay to detect the N gene, we also performed qRT-PCR on the original patient sample with the US Centers for Disease Control and Prevention (CDC) assays N1, N2 and N3 (Integrated DNA Technologies, Coralville, US) [7]. All three were positive with Ct values of 26.7, 26.2 and 26.4, respectively. 

**Figure 1:**
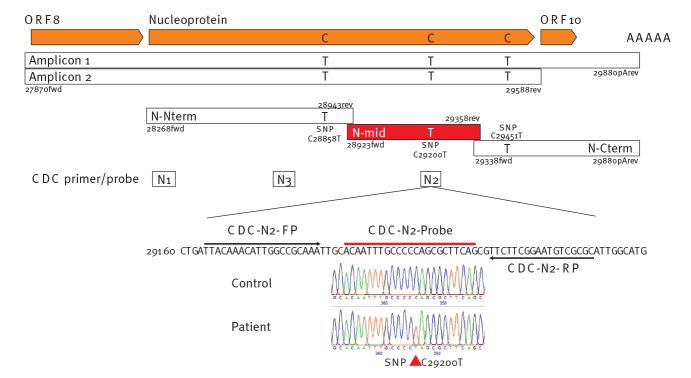
Mutations in the nucleoprotein gene of a SARS-CoV-2 patient virus and strategy to reveal the mutation responsible for loss of detection in the Xpert Xpress SARS-CoV-2 assay, Germany, July 2020

CDC: Centers for Disease Control and Prevention; Cterm: C terminus; FP: forward primer; N: nucleoprotein; Nterm: N terminus; ORF: open reading frame; RP: reverse primer; SARS-CoV-2: severe acute respiratory syndrome coronavirus 2.

The patient sample was reverse transcribed (LunaScript RT SuperMix, New England Biolabs GmbH, Frankfurt a.M., Germany) and the complete nucleoprotein (N) gene the of patient’s virus was amplified, Amplicon 1 with oligonucleotides primers 27870fwd and 29880pArev, amplicon 2 with 27870fwd and 29588rev (Table). Sanger sequencing of the two independent PCR Amplicons 1 and 2 revealed three single nucleotide polymorphisms compared with the SARS-CoV-2 strain Wuhan-HU-1 reference genome (NC_045512.2, Figure 1) [8]. 

**Table:** Oligonucleotide primers used for amplification of SARS-CoV-2 nucleoprotein gene, Germany, July 2020

Primer name	Sequence
27870fwd	GAAACTTGTCACGCCTAAACGAAC
28268fwd	ACTAAAATGTCTGATAATGGACC
28943rev	GCAGCAGCAAAGCAAGAGCAG
28923fwd	CTGCTCTTGCTTTGCTGCTGC
29358rev	GTTTTGTATGCGTCAATATGC
29338fwd	GCATATTGACGCATACAAAAC
29588rev	AGCGAAAACGTTTATATAGCCCATCTG
29880pArev	TTTTTTTTTTGTCATTCTCCTAAGAAGCTATT

SARS-CoV-2: severe acute respiratory syndrome coronavirus 2.

All PCR reactions were performed with Q5-Polymerase (New England Biolabs GmbH, Frankfurt, Germany) including GC-Enhancer. Oligonucleotide primers were obtained from Integrated DNA Technologies, Coralville, United States. Primer names reflect the 5’ end of the respective oligonucleotide on the reference genome NC_045512.2 and are used throughout Figure 1.

Three C to T conversions were detected by sequencing of patient virus amplicons 1 and 2 (position 28,858, 29,200 and 29,451 of the SARS-CoV-2 reference genome NC_045512.2, depicted in orange). Three shorter amplicons, N-Nterm, N-mid and N-Cterm, containing one single mutation each were PCR-amplified with indicated oligonucleotides (Table) to detect the responsible mutation. All positions are numbered according to the SARS-CoV-2 reference genome NC_045512.2.

Retesting of these two amplicons with the GXP assay confirmed the failure to detect the N gene from the patient virus, while the corresponding amplicon from another patient lacking these mutations was positive. Since information on the proprietary GXP N2 primers and probe(s) was unavailable, we separated the region of Amplicon 1 into three smaller independent amplicons containing only one SNP each (N-Nterm C28858T, N-mid C29200T, N-Cterm CC29451T). We amplified these from the patient virus, and as a control, from a patient lacking these SNPs. The patient’s virus amplicon N-mid, with the SNP C29200T that is within the region overlapping the CDC N2 probe, resulted in no amplification, while the same molar dilution of the N-mid amplicon of the control virus without the SNP was positive with a Ct value of 20. Therefore, the single SNP C29200T abolished detection by the N2 primer/probe set of the GXP test, while the CDC N2 primer/probe set was able to detect the patient’s virus with almost unchanged sensitivity. 

**Figure 2:**
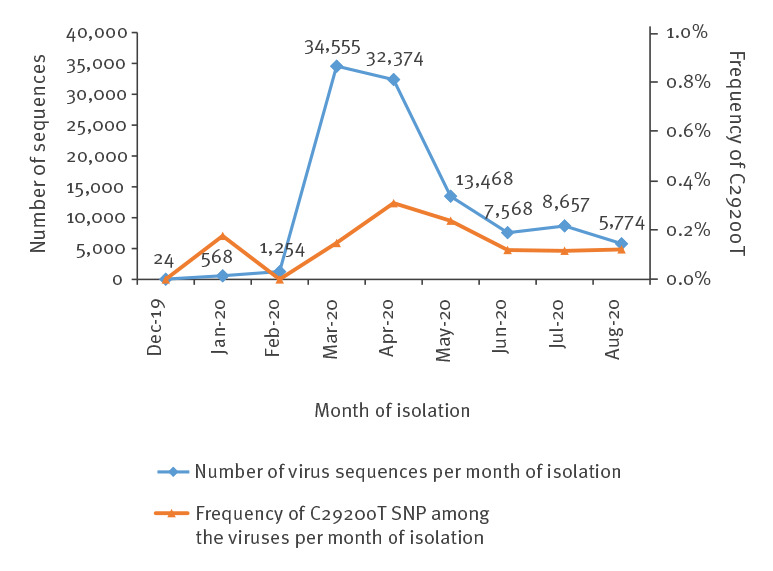
Number of SARS-CoV-2 sequences in the GISAID database and frequency of the C29200T SNP, by month of isolation, December 2019–August 2020 (n = 104,242)

SARS-CoV-2: severe acute respiratory syndrome coronavirus 2; SNP: single nucleotide polymorphism.

To estimate the frequency of strains with this SNP, we downloaded the complete SARS-CoV-2 sequences from the GISAID EpiCoV Database (release 23 September 2020, 106,572 sequences) [9] and retrieved 209 isolates (0.2%) containing this SNP C29200T by local BLASTN search [10] with CLC Genomics Workbench Version 20.0.4 (QIAGEN A/S, Aarhus Denmark) (Figure 2). The best match containing all three SNPs were three strains isolated in Romania on 12 and 13 May and 7 June. A total of 2,173 isolates had SNP C28858T (2.0%) and 96 isolates the SNP C29451T (0.1%). One sequence was isolated in Chongqing, China on 21 January 2020, and under the caveat that most sequences in GISAID sequences are from strains isolated in March and April with newer sequences only coming in, an analysis by month of isolation did not reveal apparent spreading of this synonymous variant. 

## Discussion

We could not see a link, phylogenetically or over time of isolation, between most of the strains containing the three SNPs. Only five strains had the two SNPs C28858T/C29200T and no isolate other than those from Romania had C28858T/C29451T. It is therefore likely that the SNPs were the product of spontaneous and independent mutation. The majority of interstrain SARS-CoV-2 mutations and also 60% of mutations vs the bat RaTG13 betacoronavirus genome are C-to-T transitions which may result from cytidine deamidation [11]. Isolates containing the SNP C29200T abolishing the N2 detection of the GXP assay originated from various countries including Australia, Denmark, Portugal, Saudi Arabia, the United Kingdom and the US. In the diagnostic laboratory, a SARS-CoV-2 presumptive-positive GXP result should be confirmed by another commercial or in-house test, e.g. using the published primer sets targeting the RdRp, S, or N genes [9]; our bioinformatic analysis indicates that these other N-targeting assays will not be affected by this SNP. In the absence of information on the proprietary GXP reagents, one can only speculate that the SNP may located at the 3’ end of one primer in this assay. This underlines the necessity of targeting two independent essential regions of a pathogen for reliable detection. In particular with a highly contagious agent such as SARS-CoV-2, false negative tests at hospital admission may have consequences in the clinics and for management of the pandemic.
